# A Pilot Metabolomic Study for Diagnosing *Aspergillus* Infection in Immunocompromised Pediatric Cancer Patients

**DOI:** 10.3390/ijms26135926

**Published:** 2025-06-20

**Authors:** Taghreed Khaled Abdelmoneim, Asmaa Ramzy, Mostafa Ahmed Zaki, Ahmed Karam, Ahmed Hesham, Aya Osama, Nabila Sabar, Maha Mokhtar, Nada A. Youssef, Eman A. Ahmed, Lobna Shalaby, Asmaa Salama, Ahmed Kamel, Mervat Elenany, Sameh Magdeldin

**Affiliations:** 1 Proteomics and Metabolomics Research Program, Basic Research Department, Children’s Cancer Hospital Egypt, Cairo 57357, Egypt; taghreed.khaled@57357.org (T.K.A.); asmaa.ramzy@57357.org (A.R.); mostafa.zaki@57357.org (M.A.Z.); ahmed.karam@57357.org (A.K.); amedesham9@gmail.com (A.H.); aya.osama@57357.org (A.O.); nabila.elmutassim@57357.org (N.S.); maha.muawad@57357.org (M.M.); nada.abdallah@57357.org (N.A.Y.); eman_ahmed@vet.suez.edu.eg (E.A.A.); 2 Department of Pharmacology, Faculty of Veterinary Medicine, Suez Canal University, Ismailia 41522, Egypt; 3 Infectious Diseases Department, Children’s Cancer Hospital Egypt, Cairo 57357, Egypt; lobna.shalaby@57357.org; 4 Pediatric Oncology Department, National Cancer Institute, Cairo University, Giza 12613, Egypt; 5 Pathology Department, Children’s Cancer Hospital Egypt, Cairo 57357, Egypt; dr.asmaasalama33@gmail.com; 6 Pathology Department, National Cancer Institute, Cairo University, Giza 12613, Egypt; 7 Microbiology Section, Clinical Pathology Department, Children’s Cancer Hospital Egypt, Cairo 57357, Egypt; ahmed.kamel@57357.org (A.K.); mervat.elanany@57357.org (M.E.); 8 Clinical Pathology, Faculty of Medicine, Cairo University, Giza 12613, Egypt; 9 Physiology Department, Faculty of Veterinary Medicine, Suez Canal University, Ismailia 41522, Egypt

**Keywords:** fungal infection, *Aspergillus*, pediatric cancer, metabolomics, LC-MS/MS

## Abstract

Fungal infection caused by invasive *Aspergillus* is a life-threatening complication in immunocompromised pediatric cancer patients. However, the early diagnosis of invasive infection remains a clinical challenge due to the lack of specific, non-invasive biomarkers. The current study investigates plasma metabolomic profiling integrated with an AI-derived fungal secondary metabolite database to identify potential biomarkers for rapid, non-invasive detection of Aspergillus infection. Plasma samples from thirteen pediatric oncology patients were analyzed using untargeted metabolomics based on UHPLC-MS/MS. Based on galactomannan assay results, three patients were classified as Aspergillus-Infected (AIC) and ten as non-infected controls (NPCs). An in-house custom database for secondary metabolites of fungi was incorporated to enhance metabolite annotation. Eight metabolites were found to be candidate biomarkers based on statistical significance, fold change, and biological relevance. In the AIC cohort, aflatoxin B1, aspergillimide, fumifungin, and uridine were found to be significantly elevated while citric acid presented a decrease. Multivariate analysis utilizing PCA and PLSDA showed distinct group separation. Moreover, sample size estimation indicates that a minimum of 25 participants would be needed in future studies for appropriate statistical power.

## 1. Introduction

Pediatric cancer patients face a heightened risk of opportunistic fungal infections, particularly invasive candidiasis (IC) and invasive aspergillosis (IA), due to prolonged immunosuppression from chemotherapy and stem cell transplantation [1]. Invasive fungal infections (IFIs) are a major cause of morbidity and mortality in this vulnerable population. Global estimates suggest at least 13 million infections and over 1.5 million annual deaths. Mortality rates can be up to 26 times higher due to treatment-induced acute kidney injury, which significantly worsens IFI outcomes [2,3].

Early diagnosis is critical; however, conventional methods such as microscopy and culture are invasive and slow, counting for 3–5 days. Biomarker-based approaches, including the galactomannan (GM) test, offer faster and more sensitive diagnostics. However, the GM test has several important limitations that can affect its diagnostic sensitivity, such as false negatives, false positives, and its lowered sensitivity in serum samples compared to bronchoalveolar lavage (BAL) fluid. Consequently, it is clinically preferrable to use BAL fluid instead of blood, leading to more invasive procedures [4,5]. Furthermore, GM has been utilized to support the diagnosis of invasive aspergillosis (IA) and to monitor and predict patient outcomes [6]. Across various studies, the reported sensitivity of the GM test has ranged widely, from 57% up to 100% [7].

Metabolomics has emerged as a powerful tool for fungal infection diagnosis by identifying metabolic alterations that occur during pathogen–host interactions [8]. These metabolic changes arise from both fungal metabolic activity and the host’s immune response, providing valuable biomarkers for disease detection. Metabolomics focuses on analyzing small-molecule metabolites present in biofluids such as blood, urine, and cerebrospinal fluid. Fungal infections induce distinct metabolic shifts, including alterations in lipid metabolism, oxidative stress responses, and energy production pathways. Additionally, fungal pathogens secrete unique metabolites, some of which can serve as a potential indicators of infection [9]. By comparing metabolic profiles of infected and non-infected individuals, researchers can identify disease-specific signatures that enable early and accurate diagnosis. Novel metabolites such as 3-indolealdehyde (3-IAld) and versicolorin B have demonstrated potential in differentiating active infection from mere colonization, addressing a critical gap in current diagnostic methodologies [10]. These advancements pave the way for improved patient management, earlier therapeutic interventions, and more effective antifungal stewardship.

Liquid Chromatography–Mass Spectrometry (LC-MS/MS) is a cornerstone of metabolomics-based diagnostics due to its high sensitivity, broad metabolite coverage, and ability to detect low-abundance compounds [11]. LC-MS/MS offers greater analytical resolution by separating complex biological mixtures and providing detailed molecular characterization. Its flexibility in detecting a wide range of metabolites, including lipids, amino acids, and organic acids, makes it an essential tool in fungal infection diagnostics [12]. Advancements in high-resolution mass spectrometry and data processing further enhance its capability to differentiate subtle metabolic variations associated with infections, improving diagnostic accuracy and clinical decision-making.

However, the application of metabolomics in fungal diagnostics presents challenges, including sample variability, metabolite instability, and complex data interpretation. Standardizing metabolomics workflows and improving computational tools for biomarker discovery are crucial for translating research findings into clinical practice [13]. Taken together, metabolomics can enhance diagnostic capabilities and ultimately reduce IFI-related mortality and the associated economic burden.

Artificial intelligence (AI) is the ability of machines to perform tasks that normally require human intelligence, such as language processing, pattern recognition, finding hidden connections, and complex data summarization. On the other hand, machine learning (ML) is a subfield of AI that uses algorithms to learn from data and automatically improve its performance [14]. In the context of mass spectrometry, AI and ML methods can deconvolute overlapping peaks in complex chromatograms and improve peak selection [15,16]. In metabolomics, both are implemented to normalize and integrate large datasets in order to overcome batch effect [17]. Additionally, they are extensively used for feature selection and classification, utilizing supervised and unsupervised learning methods such as partial least square discriminant analysis (PLS-DA) and principal component analysis (PCA) to manage a large number of variables in metabolomics datasets and highlight metabolites associated with the phenotype or condition of interest [18].

Beyond improving analytical performance, AI enhances data reproducibility by reducing human biases in manual interpretation. While traditional workflows rely on expert-driven curation, AI-driven models offer a standardized and efficient approach to metabolomics analysis [19]. However, challenges persist, including the need for well-annotated datasets, computational infrastructure, and the interpretability of AI-generated findings. Advancing AI-driven pipelines will strengthen the clinical application of metabolomics, accelerating its impact on personalized medicine and infectious disease management [19,20].

This pilot study aims to identify candidate metabolites that may serve as non-invasive candidate biomarkers for distinguishing patients with fungal infections. By analyzing the metabolic profiles associated with fungal infections, the detection of blood-leached fungal metabolites could improve diagnostic accuracy and reduce the time required for diagnosis. In addition, advancing the fungal metabolomics research by creating a specialized, high-resolution database of fungal secondary metabolites powered by AI would facilitate ongoing fungal research toward precise candidate biomarker annotation. Despite its limited sample size, this pilot study is a crucial precursor for a larger prospective study.

## 2. Results

### 2.1. Study Subjects

In this study, 13 pediatric cancer patients were divided into two cohorts: those with confirmed *Aspergillus* infections (Aspergillus-Infected Cancer (AIC), *n* = 3) and those without fungal infections (Non-Fungal Pediatric Cancer (NPC), *n* = 10). The mean age of participants in the AIC and NPC cohorts was 11.6 ± 6.6 and 7 ± 3.3 years, respectively (Table 1). The AIC cohort consisted of 3 female patients, while the NPC cohort included 4 male and 6 female patients. All participants were diagnosed with cancer. The AIC cohort included 2 patients with acute lymphoblastic leukemia (ALL), and 1 with Wilm’s tumor (WT). The NPC cohort included 3 patients with acute myeloid leukemia (AML), 3 with ALL 2 with WT, 1 with neuroblastoma (NB), and 1 with lymphoma.

### 2.2. Sample Collection and Preparation

The plasma metabolomics of AIC and NPC cohorts were screened to determine the differentially expressed metabolites (DEMs) that differentiate between the two cohorts using the LC-MS/MS. The primary search for both the positive and negative modes generated a total of 556 fungal-derived metabolic features across all samples. After pre-processing and normalization (ppm error cut-off, removing duplicates, and manual curation), 56 fungal-derived metabolites had passed the analysis criteria (Table 2). Substantially, 41 metabolites were relatively quantified (shared) in the two cohorts. Eight metabolites were uniquely determined in the AIC cohort (did not pass the threshold in counterpart group), while seven metabolites were uniquely determined in NPC (as mentioned in Table 2).

### 2.3. Univariate Statistical Analysis

A comprehensive statistical analysis of the metabolomics dataset was conducted in order to identify metabolic differences between AIC and NPC cohorts. The significantly changed metabolites were found using both univariate and multivariate methods to highlight the unique metabolic signatures linked to *Aspergillus* infections. The normality of the metabolomic data was evaluated using the Shapiro–Wilk test. This test quantitatively evaluates whether a sample comes from a normally distributed population, with the resulting *p*-values ≤ 0.05 suggesting a significant deviation from normality. Furthermore, auto-scaling was applied to standardize the metabolomic dataset prior to statistical analysis. The Wilcoxon Mann–Whitney test, a non-parametric statistical test, was employed to compare metabolite concentrations between cohorts and identify DEMs. To further refine the selection of DEMs, additional thresholds were applied, including a Log2 fold change (FC) ≥ 1 or ≤−1 and an adjusted *p*-value ≤ 0.05. The adjusted *p*-value was calculated using the false discovery rate (FDR) method to control for multiple comparisons and reduce the risk of false positives.

**Figure 1:**
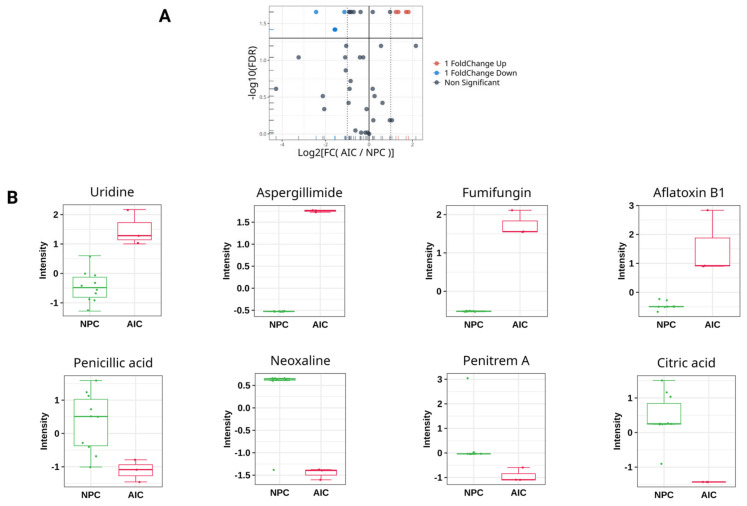
Univariate analysis for the plasma metabolomics profile of the Aspergillus-Infected Cancer (AIC) cohort compared to Non-Fungal Pediatric Cancer (NPC) cohort. (**A**) Volcano plot showing the log2 (fold change) between the Aspergillus-Infected Cancer (AIC) and Non-Fungal Pediatric Cancer (NPC) (*y*-axis), and −log10 (FDR) calculated by Wilcoxn rank-sum test to show the significant DEMs. The red color represents DEMs that are significantly upregulated (FDR ≤ 0.05, log2 (FC) ≥ 1), while the blue color represents the significant downregulated DEMs (FDR ≤ 0.05, log2 (FC) ≤ −1). The grey color represents metabolites that are not significant. (**B**) Box plot representation with auto-scaled intensities for the significant DEMs (FDR ≤ 0.05, log2 (FC) ≥ 1 or ≤−1). The green color represents samples from the NPC cohort, and red color represents samples from the AIC cohort.

The analysis revealed eight significant DEMs (FDR ≤ 0.05, log2 (FC) ≥ 1 or ≤−1) between the studied cohorts (Figure 1), indicating metabolic perturbations due to *Aspergillus* infection (Table 3). Four metabolites were downregulated in the AIC cohort compared to the NPC cohort. These metabolites are as follows: citric acid, penicillic acid, neoxaline, and penitrem A. Meanwhile, four metabolites were upregulated in the AIC cohort compared to the NPC cohort. These metabolites are uridine, fumifungin, aflatoxin b1, and aspergillimide. Of note, aspergillimide and citric acid showed relatively significant log2 (FC) values of 1.8 and −2.4, respectively.

### 2.4. Multivariate Statistical Analysis

**Figure 2:**
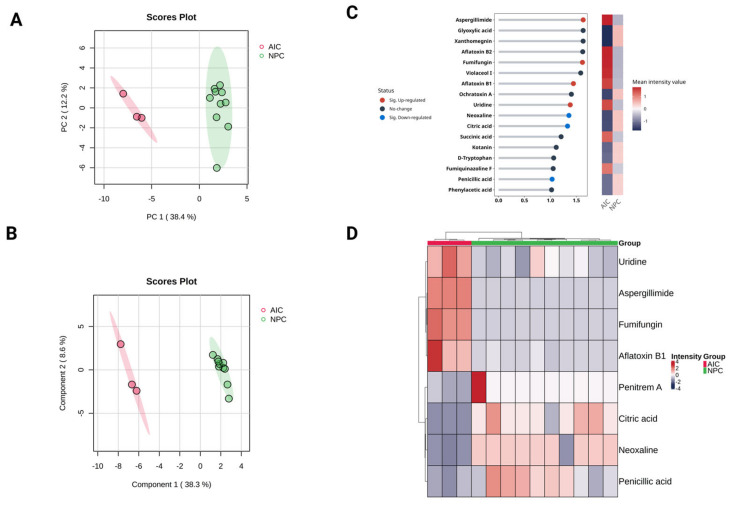
Multivariate analysis for the plasma metabolomics profile of the Aspergillus-Infected Cancer (AIC) cohort compared to the Non-Fungal Pediatric Cancer (NPC) cohort. Metabolite intensities were auto-scaled before the analysis. (**A**) Principal component analysis (PCA) and (**B**) partial least square discriminant analysis (PLS-DA) for the metabolomics profile, respectively. (**C**) Variable importance in projection (VIP) score plot for the AIC and NPC cohorts displays the top 17 most important metabolite features identified by PLS-DA (VIP score ≥ 1). The colored heatmap on the right indicates the relative concentration of corresponding metabolites for the AIC and NPC cohorts. The blue dots for each horizontal line represent metabolites that are significantly downregulated DEMs (FDR ≤ 0.05, log2 (FC) ≤ −1), while red dots are significantly upregulated DEMs (FDR ≤ 0.05, log2 (FC) ≥ 1). (**D**) The hierarchical cluster analysis (HCA) heatmap plot highlights the significant DEMs (FDR ≤ 0.05, log2 (FC) ≥ 1 or log2 (FC) ≤ −1).

Unsupervised multivariate PCA was applied to inspect the potential differences between the AIC cohort and the NPC cohort based on their metabolomic profiles. The resulting score plot showed clear separation along Principal Component 1 (PC1), which captured 38.4% of the total variance (Figure 2). There is minimal overlap, indicating a clear metabolic distinction between the two cohorts. PC2 accounts for 12.2% of the variance, showing some variability within each cohort but not contributing as much to their separation. The NPC cohort exhibits more spread along PC2, suggesting higher intra-group variability compared to the AIC cohort. Furthermore, PLS-DA was applied to identify the most important features (metabolites) contributing to classification. PLS-DA is a supervised method that maximizes the separation between groups and generally improves discrimination, making it more useful for biomarker discovery. Similar to PCA, the PLS-DA showed a clear separation between the two cohorts. Accuracy and permutation tests confirmed that the observed clustering was statistically significant (Figure 2).

To hasten the evaluation of the key metabolites contributing to the differentiation between the AIC and NPC cohorts, VIP scores from the PLS-DA model were assessed. VIP scores rank metabolites based on their contribution to the model. The greater the score is, the more it indicates its role in the discrimination between the cohorts. Generally, metabolites with VIP scores > 1 are considered significant contributors to separation. The VIP analysis identified 17 key metabolites, many of which overlap with the significant DEMs reported in the univariate analysis (Figure 2). The VIP analysis supports the findings from univariate statistical analysis, confirming that uridine, aspergillimide, fumifungin, and aflatoxin B1 are key differentiating metabolites.

Additionally, a hierarchical clustering heatmap was generated based on the DEMs to explore the metabolic differences between the two cohorts. The heatmap reveals clear segregation between the AIC (disease) and NPC (control) groups, indicating significant metabolic alterations due to *Aspergillus* infection (Figure 2). The AIC samples (red group) appear to cluster more tightly, indicating a more uniform metabolic response to *Aspergillus* infection. The NPC samples (green group) exhibit a wider spread, suggesting higher intra-group variability in metabolomics profiles.

### 2.5. Sample Size 

**Figure 3:**
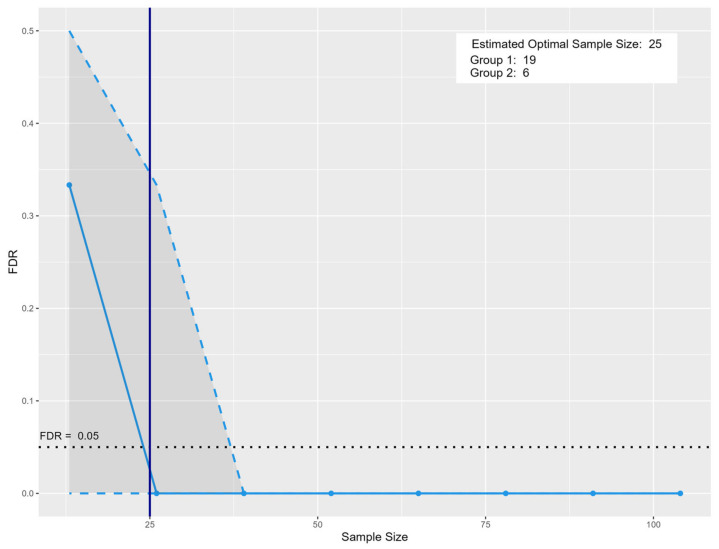
Sample size estimation for prospective metabolomics-based biomarker discovery. The solid blue vertical line indicates the recommended sample size of 25, with 19 samples in Group 1 and 6 in Group 2. The blue solid line represents the estimated false discovery rate (FDR) at varying total sample sizes. The dashed blue line represents the FDR trend across varying sample sizes, showing a sharp decline as the sample size increases. The vertical solid blue line marks the estimated optimal total sample size (*n* = 25), comprising 19 subjects in Group 1 and 6 in Group 2, at which point the FDR falls below the 0.05 threshold. The black dotted line marks the targeted 0.05 FDR threshold. The shaded region highlights areas of higher uncertainty in estimation at smaller sample sizes.

The analysis was conducted using this pilot dataset, incorporating the following parameters: *type.exp* = “Targeted”; *prop.sig* = 0.1428, representing the proportion of significant metabolite features identified in this study dataset (significant metabolites = 8 divided by the total obtained metabolite features = 56); *model* = “PPCA”; and *n.per.group* = 10 for NPC and 3 for AIC, reflecting the number of samples per group in this study dataset. Additionally, a target FDR of 0.05 was set to ensure robust statistical confidence. Based on this simulation-based sample size estimation using the MetSizeR package (version 2.0.0), a minimum of 25 participants (NPC = 19, and AIC = 6) is justified to maintain a controlled false discovery rate (FDR) at 0.05 while preserving statistical power (Figure 3). This sample size provides a balance between minimizing Type I errors and ensuring adequate sensitivity to detect true associations within the dataset. This justification supports the robustness of the chosen sample size in ensuring reliable statistical inference in a high-dimensional multiple testing context.

## 3. Discussion

Our study sought to identify candidate biomarkers for the reliable detection of *Aspergillus* infection in immunocompromised pediatric cancer patients. However, this pilot study involved a limited sample size, which may constrain the generalizability of the findings. It is worth noting that recruiting immunocompromised pediatric cancer patients with confirmed *Aspergillus* infections who also meet stringent inclusion criteria is particularly challenging due to the complexity of their clinical conditions. Also, the limited cancer patients’ numbers that exhibit a clear systemic invasive *Aspergillus* infection without other complications (apart from cancer). Nevertheless, this study lays the groundwork for larger-scale validation. The primary goal was to generate initial data for a prospective large-scale research. Taken together, this pilot successfully demonstrated the potential of combining metabolomics and artificial intelligence (AI) for diagnosing fungal infections in children with cancer.

This research significantly advanced fungal metabolomics by creating a specialized, high-resolution spectral database for fungal secondary metabolites powered by AI. This database addresses the inherent difficulties of analyzing vast spectral datasets and the inadequacies of current public databases. To achieve this, we employed artificial intelligence, developed with custom R scripts, to automate spectral data collection, validation, and integration, resulting in more accurate fungal-derived metabolite identification.

Based on the availability of participants that match the study inclusion/exclusion criteria, we collected plasma samples from 13 cancer patients. *Aspergillus* infection was identified in 3 patients (AIC cohort) using sera galactomannan test, while the remaining 10 patients (NPC cohort) served as negative controls. Patients with positive blood cultures for bacterial infection were excluded, as bacterial infection is a main confounder for the study objective. We hypothesized that untargeted metabolite profiling, using UHPLC-MS/MS in conjunction with specific in-house fungal database, could identify novel candidate biomarkers of fungal infection. This approach, we proposed, would enable a more accurate diagnosis and contribute to reducing the unnecessary prescription of prophylactic antifungal drugs.

Following the initial analysis of the UHPLC-MS\MS spectra, 41 metabolites were found to be shared in both groups. The presence of fungal-associated metabolites in non-infected patients may result from dietary exposure to mycotoxins [21], environmental contamination [22], or secondary metabolite production by commensal microbiota [23]. Further studies with dietary, environmental, and microbiome profiling are needed to distinguish background fungal-derived metabolites from infection-specific biomarkers.

These metabolites were subsequently filtered down to 8 metabolites based on 3 filtration criteria: (i) statistical significance, (ii) fold change, and (iii) biological relevance. Four of these metabolites were upregulated in AIC including aflatoxin B1, aspergillimide, fumifunginin, and uridine. On the other hand, citric acid was a significantly downregulated metabolite in AIC, with more biological relevance to our study than the remaining 3 downregulated metabolites.

Regarding the upregulated metabolites in AIC, aflatoxins are groups of mycotoxins that are known to be carcinogenic. They are extracted from *Aspergillus flavus* (*A. flavus* toxin), with aflatoxin B1 (AFB1) being the most potent one that causes hepatocellular carcinoma. Inside the human body, specifically the liver, AFB1 is oxidized into aflatoxin B1-8,9-epoxide (AFBO), which possesses high electrophilicity, allowing it to react with amino acids and nitrogenous bases of the nucleic acid, spontaneously making it carcinogenic [24]. Aspergillimide, also known as asperparaline A, were identified and isolated from *Aspergillus japonicus* JV-23; it also possesses paralytic properties as it causes paralysis of silkworm larvae [25]. Thus, it has been used as an antiparasitic drug [26]. Fumifungin is a mycotoxin known to be produced by *A. fumigatus*. It also possesses antifungal activity against several fungal species [27]. In addition, it was detected as a virulence factor that is only produced by *A. fumigatus* from clinical isolates exclusively [28]. Uridine, a molecule composed of uracil nitrogenous base and ribose sugar, is important for fungal RNA and cell wall synthesis. Previous studies states that uridine auxotrophic *Aspergillus fumigatus* infecting murine model of aspergillosis exhibits decreased virulence and exposed cell wall immunogenic polymers making it nonpathogenic. However, when drinking water supplemented with uridine are provided, the fungi restored its pathogenicity [29,30].

The downregulation of citric acid in AIC patients could be due to its consumption during the tricarboxylic acid (TCA) cycle for energy production by immune cells during inflammation. In addition, citric acid is catabolized to produce acetyl-CoA, a key compound for fatty acid synthesis pathway that is reported to have a key role in cell proliferation and membrane expansion, which is essential for the activation of dendritic cells, natural killer cells, and the differentiation of M1 macrophage. In addition, they are essential for membrane-bound organelle synthesis and cell signaling during immune response [31,32].

Among the identified metabolites, aspergillimide is the highest upregulated metabolite with an approximately 3.5 times growth in AIC patients, whereas citric acid is the most relevant downregulated metabolite where its occurrence is reduced to approximately one-fifth from NPC to AIC, strongly suggesting their usage as a candidate biomarker of aspergillosis. Other *Aspergillus* mycotoxins, aflatoxin B1 and fumifungin, are also strong candidates as their fold change expression were approximately 3 and 2.5 times higher in AIC patients, respectively. In addition, they had the highest VIP score among the identified metabolites as well as uridine, which had 2.3 times increased expression fold change in AIC patients. The PCA and PLS-DA analysis of the obtained metabolic profiles of AIC and NPC showed that both groups are separated and clustered together. Markedly, the PCA showed that NPC cohort exhibits more spread along PC2, suggesting higher intra-group variability compared to the AIC cohort. This may be due to the high variability of cancer types in the NPC cohort, so we suggest including less variant cancer types and more stringent inclusion criteria in the prospective study.

Based on sample size estimation, the results suggest that a minimum of 25 participants (NPC = 19, AIC = 6) are required to achieve adequate statistical power while controlling for false discoveries. This sample size optimization ensures that future studies can maintain sufficient sensitivity to detect true metabolomics associations while mitigating the risks of false-positive findings.

## 4. Materials and Methods

### 4.1. Study Participants

In this study, 13 pediatric cancer patients were divided into two cohorts: those with confirmed *Aspergillus* infections (Aspergillus-Infected Cancer (AIC), *n* = 3) and those without fungal infections (Non-Fungal Pediatric Cancer (NPC), *n* = 10). Plasma samples were collected from Clinical Pathology Department, Microbiology Unit at Children’s Cancer Hospital 57357, Egypt (CCHE-57357), after Institutional Research Board approval (IRB-CCHE-57357-17-2020). An informed consent was signed by the guardians of the patients prior to sample collection. The AIC cohort were patients with confirmed malignancies who were 18 years or younger and who also had a positive galactomannan antigen (index > 0.5) in serum. The NPC cohort were patients undergoing maintenance chemotherapy who were not neutropenic or febrile, did not have any pulmonary disease, and had negative galactomannan antigen (index < 0.5) in serum.

Criteria for exclusion were the presence of uncontrolled infections, uncontrolled psychiatric or neurological diseases, known active HBV, HCV, or Hepatitis A infections, or bleeding disorders not related to cancer. Also, patients with positive blood cultures for bacterial infection were excluded. Both male and female subjects were included in the study. The total demographic data of the studied cohorts are presented in Table 1. All the samples collected were kept at −80 °C until use.

### 4.2. Reagents and Chemicals

Acetonitrile and ammonium formate 99% were obtained from Sigma-Aldrich (Sigma-Aldrich Co., St. Louis, MO, USA), while methanol, chloroform, formic acid, and sodium hydroxide were obtained from Thermo-Fisher (Thermo-Fisher Scientific, Loughborough, UK). Moreover, the water was obtained from Thermo-Fisher (Thermo-Fisher Scientific, Waltham, MA, USA). All solvents are of HPLC grade.

### 4.3. Sample Pre-Processing for Metabolomics Analysis

Thirteen plasma samples were thawed, aliquoted into 50 µL and exposed to metabolite extraction. In brief [33], every sample was exposed to 300 μL of precooled extraction solvent made up of chloroform: water: methanol with ratios 1:1:3, respectively. It was whirled and vortexed for at least 2 min at approximately 20 °C, ultrasonicated (Elma Elmasonic Ultrasonic cleaning unit P, Elma, Germany) for 15 min at 4 °C, and centrifuged (Tomy MX-207 High-Speed Refrigerated Micro Centrifuge, Tomy Kogyo Co., Ltd., Tokyo, Japan) at 12,000 rpm at 4 °C for 10 min.

The samples were dried afterwards using a SpeedVac (Concentrator plus Eppendorf, Hamburg, Germany) at 30 °C and reconstituted in a solvent system of water, methanol, and acetonitrile in the ratio 2:1:1, respectively. The extracts were separated using Liquid Chromatography with tandem mass spectrometry (LC-MS/MS) [34].

For the quality control samples, equal quantities of 15 µL of all samples were mixed to create quality control QCs (QC-pool); equal volumes of 30 µL of samples from positive group (QC-AIC); and equal volumes of 30 µL of samples from negative group (QC-NPC). Mass calibration was performed every 2 h using positive or negative APCI calibration solution (AB SCIEX) through an automated calibration delivery system. Blank samples were used to remove any potential carryover and to determine the quality of all runs.

### 4.4. Analysis of UHPLC-MS/MS Using Information-Dependent Acquisition (IDA)

Information-Dependent Acquisition (IDA) was used for LC-MS/MS analysis of the samples acquired in positive and negative ionization modes [35]. ExionLC™ AC UHPLC system (AB SCIEX, Concord, ON, Canada) with Acquity XSelect HSS T3 analytical column 2.1 × 150 mm, 2.5 µm (Waters Co., Milford, MA, USA) combined with Triple TOF 5600+ mass spectrometer (AB SCIEX, Concord, Canada) was used for analysis.

For the chromatographic separation, 10 µL of each of the sample was injected within 35 min by gradient elution with a flow rate of 0.3 mL/min. The mobile phase solutions were as follows: solution (A), 5 mM ammonium formate in 1% methanol (pH 3.0) for elution in positive mode; solution (B), 5 mM ammonium formate in 1% methanol (pH 8.0) for elution in negative mode; solution (C), acetonitrile 100%. Gradient elution was also programmed as 0% C for 1.0 min, 0–90% C in 20 min, 90% for 4.0 min, 90–0% C in 1.0 min, and finally 3.0 min re-equilibration using 0% C. Both positive-ion (ESI+) and negative-ion (ESI-) modes of mass spectrometric detection were operated in a DuoSprayTM ion source [33,34].

IDA with dynamic background subtraction was utilized to identify metabolites in the samples by having TOF-MS scan from 50 to 1000 Da gathered in 30 ms, followed by MS/MS of the 15 most intense precursor ions from 50 to 1000 Da with a fixed 50 Da transition window. The accumulation time for both MS/MS acquisitions was 50ms, and the collision energies were 35 V and −35 V for positive and negative mode, respectively. The cycle time was 0.6502 s. MS and MS/MS spectra were recorded using Analyst TF (v 1.7.1) [33].

### 4.5. Development of a Metabolite Identification Database

To streamline the processing of extensive datasets generated in untargeted fungal metabolomics experiments, we developed in-house R scripts (version 4.2.2) for spectral data retrieval and validation (unpublished data) as performed in our previous work on colorectal cancer [36], utilizing the DELLEMC PowerEdge R760 server throughout. This resulted in the creation of a high-resolution, in-house database of fungal secondary metabolites (Supplementary Files S1 and S2). To ensure data consistency and relevance, several key modifications were incorporated, such as the sole use of experimental LC-MS data for method consistency, selection of specific adduct types for improved accuracy, and limitation of the dataset to endogenous fungal secondary metabolites. Based on the previously published data regarding the fungal secondary metabolites and artificial intelligence (AI) assistance, a specific database for the fungi was built [37,38,39]. The fungal annotation pipeline used in this study was developed in R and includes spectral matching against an in-house fungal secondary metabolite database. The R script is available at: https://gitlab.com/prolab11/fungal-secondary-metabolite-spectral-library (accessed on 15 June 2025). This focused strategy refined the dataset by prioritizing those metabolites most applicable to fungal biology and pathogenesis and improving the accuracy of fungal metabolomics profiling. The curated metabolite data also facilitated the additional investigation of fungal secondary metabolite-associated metabolic processes, which resulted in the determination of major pathways and metabolites implicated in fungal development, interaction, and pathogenicity.

### 4.6. Metabolomics Data Analysis

Metabolite identification was carried out using the MS-DIAL 4.9.2 software [40]. The in-house constructed database served as a search space. To give greater confidence to the identified metabolites at parents and fragment ions levels [40], manual verification was carried out using PeakView 2.2 with MasterView 1.1 program (AB SCIEX) packages [34]. Meanwhile, search parameters—precursor ion XIC S/N > 10, sample: blank > 5—and precursor mass tolerance 10 ppm were used. Obtained output was treated with statistical analysis and biological screening [33].

### 4.7. Data Pre-Processing and Statistical Analysis

All pre-processing, statistical analysis, and visualization were performed using R (version 4.2.2) [41]. MS-DIAL output was pre-processed [40] using probabilistic quotient normalization (PQN) [42] and filtering of duplicates (keeping the highest intensity feature). Missing values were imputed randomly within ±10% of the group’s median [43], and auto-scaling was applied [44]. Normality was tested with the Shapiro–Wilk test (*p* ≤ 0.05) [45]. Differentially significant metabolites (DEMs) were identified using the Wilcoxon Mann–Whitney test (Log_2_ fold change (FC) ≥ 1 or ≤−1, FDR-adjusted *p*-value ≤ 0.05) [46]. Multivariate analysis included principal component analysis (PCA), partial least square discriminant analysis (PLS-DA), and hierarchical clustering analysis (HCA).

### 4.8. Sample Size Estimation

To determine the optimal sample size for detecting significant metabolic differences in pediatric cancer patients with and without fungal infections, we employed the MetSizeR package in R (version 2.0.0) [47] based on study-specific parameters. This package, designed for metabolomics, estimates sample size based on pilot data variability while controlling the false discovery rate (FDR). The *MetSizeR.estimate.samplesize()* function, with its built-in plotting, determined the sample size at the point where the FDR (0.05) was met with sufficient power. Key parameters included the following: *type.exp* (“Untargeted” for our exploratory analysis), *prop.sig* (proportion of significant features from pilot data), *model* (PPCA simulation), and *n.per.group* (pilot study sample size per group).

## 5. Conclusions

In conclusion, this study represents preliminary data due to the limited sample size, serving as an essential proof of concept. The recruitment of subjects was challenging due to the complex clinical profiles of immunocompromised pediatric patients and the stringent inclusion/exclusion criteria required to eliminate confounding that may hinder the study outcomes. While it lays the groundwork for future large-scale research, caution must be exercised when interpreting these findings. This study also introduces a specialized fungal secondary metabolite spectral database, which successfully improved the specificity of metabolite annotation in complex biological samples. Multivariate analyses such as PCA and PLS-DA demonstrated clear clustering between the AIC and NPC groups, highlighting distinct metabolic profiles despite the small cohort size. Moving forward, an expanded cohort, longitudinal sampling, and multi-omics integration will be necessary to strengthen the clinical utility of plasma fungal metabolomics in infection diagnostics. Future studies should aim for larger patient groups while adhering to consistent inclusion/exclusion criteria to enhance reproducibility and validate biomarkers more effectively. Despite these constraints, this study is considered an important first step toward unraveling the metabolomics landscape of *Aspergillus* infections in pediatric cancer patients.

## Figures and Tables

**Table 1:** Baseline demographic and patient’s related information.

	Non-Fungal Pediatric Cancer (NPC)	Aspergillus-Infected Cancer (AIC)
Age (Mean, ±SD)	7 ± 3.3	11.6 ± 6.6
Males (N)	4	NA
Females (N)	6	3
Type of cancer (N)	3 ALL 2 WT 3 AML 1 NB 1 Lymphoma	2 ALL 1 WT

N = number of participants; ALL: acute lymphoblastic leukemia; WT: Wilm’s tumor; AML: acute myeloid leukemia; NB: neuroblastoma.

**Table 2:** A list of the relatively quantified metabolites and their fold change and adjusted *p*-value.

Metabolite Name	log2 (FC)	*p*. Adjusted	Status
Citric acid *	−2.44	0.022	Relatively quantified in both cohorts
Aspergillimide *	1.82	0.022	Relatively quantified in both cohorts
Aflatoxin B1 *	1.71	0.022	Relatively quantified in both cohorts
Fumifungin *	1.35	0.022	Relatively quantified in both cohorts
Uridine *	1.24	0.022	Relatively quantified in both cohorts
Penitrem A *	−1.13	0.022	Relatively quantified in both cohorts
Aflatoxin B2	0.97	0.022	Relatively quantified in both cohorts
Kotanin	−0.91	0.022	Relatively quantified in both cohorts
Phenylacetic acid	−0.89	0.022	Relatively quantified in both cohorts
Glyoxylic acid	−0.82	0.022	Relatively quantified in both cohorts
Ochratoxin A	−0.71	0.022	Relatively quantified in both cohorts
Xanthomegnin	−0.39	0.022	Relatively quantified in both cohorts
Violaceol I	0.17	0.022	Relatively quantified in both cohorts
Penicillic acid *	−1.58	0.038	Relatively quantified in both cohorts
Neoxaline *	−1.56	0.038	Relatively quantified in both cohorts
Succinic acid	2.16	0.064	Relatively quantified in both cohorts
D-Tryptophan	−1.05	0.064	Relatively quantified in both cohorts
Rubrofusarin	0.55	0.064	Relatively quantified in both cohorts
4-Hydroxybenzaldehyde	−3.24	0.091	Relatively quantified in both cohorts
Indole	−1.10	0.091	Relatively quantified in both cohorts
Caproic acid	−0.42	0.091	Relatively quantified in both cohorts
Kojic acid	−0.27	0.091	Relatively quantified in both cohorts
Itaconic acid	−1.07	0.137	Relatively quantified in both cohorts
Citrinin	−0.85	0.191	Relatively quantified in both cohorts
Malformin	−4.28	0.244	Relatively quantified in both cohorts
fumagillin	−0.90	0.244	Relatively quantified in both cohorts
Silybin B	0.17	0.244	Relatively quantified in both cohorts
Mycophenolic acid	−2.13	0.306	Relatively quantified in both cohorts
Gentisyl alcohol	0.25	0.306	Relatively quantified in both cohorts
Emodin	−0.94	0.379	Relatively quantified in both cohorts
Fumiquinazoline F	0.62	0.379	Relatively quantified in both cohorts
Sulochrin	−2.06	0.460	Relatively quantified in both cohorts
Warfarin	−0.11	0.460	Relatively quantified in both cohorts
Lovastatin acid	1.05	0.653	Relatively quantified in both cohorts
Fumigaclavine A	0.96	0.653	Relatively quantified in both cohorts
Viridicatin	0.20	0.653	Relatively quantified in both cohorts
Cyclopeptine	−0.63	0.899	Relatively quantified in both cohorts
Fumigaclavine C	−0.36	0.960	Relatively quantified in both cohorts
Aflatoxin G	−0.17	0.960	Relatively quantified in both cohorts
Imperatorin	−0.07	0.960	Relatively quantified in both cohorts
Dehydrocyclopeptine	0.01	1	Relatively quantified in both cohorts
Taichunamide H	NA	NA	Uniquely quantified in NPC cohort
6,8-dihydroxy-3-methylisocoumarin	NA	NA	Uniquely quantified in NPC cohort
Aflatoxin G2	NA	NA	Uniquely quantified in NPC cohort
Orcinol	NA	NA	Uniquely quantified in NPC cohort
Oxaline	NA	NA	Uniquely quantified in NPC cohort
Silybin B	NA	NA	Uniquely quantified in NPC cohort
Viridicatumtoxin	NA	NA	Uniquely quantified in AIC cohort
Dihydroaflatoxin G1	NA	NA	Uniquely quantified in AIC cohort
Asterric acid	NA	NA	Uniquely quantified in AIC cohort
Chrysophanol	NA	NA	Uniquely quantified in AIC cohort
Cyclopenol	NA	NA	Uniquely quantified in AIC cohort
Physcion	NA	NA	Uniquely quantified in AIC cohort
Sterigmatocystin	NA	NA	Uniquely quantified in AIC cohort
Trypacidin	NA	NA	Uniquely quantified in AIC cohort

* Represents statistical significance at FDR ≤ 0.05 and log2 (FC) ≥ 1 or log2 (FC) ≤ −1. NA; Not applicable (no statistical analysis done).

**Table 3:** Significantly differential expressed metabolites (DEMs) in the Aspergillus-Infected Cancer (AIC) cohort compared to Non-Fungal Pediatric Cancer (NPC) cohort.

Metabolite Name	log2 (FC)	*p*. Ajusted	Regulation
Citric acid	−2.438	0.022	Downregulated
Penicillic acid	−1.585	0.038	Downregulated
Neoxaline	−1.555	0.038	Downregulated
Penitrem A	−1.133	0.022	Downregulated
Uridine	1.237	0.022	Upregulated
Fumifungin	1.351	0.022	Upregulated
Aflatoxin B1	1.708	0.022	Upregulated
Aspergillimide	1.816	0.022	Upregulated

## Data Availability

The original contributions presented in this study are included in the article/Supplementary Materials. Further inquiries can be directed to the corresponding authors.

## Supplementary Materials

The following supporting information can be downloaded at: https://www.mdpi.com/article/10.3390/ijms26135926/s1.
