# Supplementary material for: A Pilot Metabolomic Study for Diagnosing Aspergillus Infection in Immunocompromised Pediatric Cancer Patients
Source: Int J Mol Sci. 2025 Jun 20;26(13):5926. doi: 10.3390/ijms26135926 (PMC12249737; doi:10.3390/ijms26135926)
Supplement: Supplementary file 1 [file ijms-26-05926-s001.zip › Supplementary/Supplementary File Name.pdf]

File S1: This file contains the in-house curated fungal secondary metabolite database in MSP format, developed for the positive ionization mode.

File S2: This file contains the in-house curated fungal secondary metabolite database in MSP format, developed for the negative ionization mode
